# Global Metabolomics Reveals Urinary Biomarkers of Breast Cancer in a MCF-7 Xenograft Mouse Model

**DOI:** 10.3390/metabo3030658

**Published:** 2013-08-07

**Authors:** Caroline H. Johnson, Soumen K. Manna, Kristopher W. Krausz, Jessica A. Bonzo, Raymond D. Divelbiss, Melinda G. Hollingshead, Frank J. Gonzalez

**Affiliations:** 1 Laboratory of Metabolism, Center for Cancer Research, National Cancer Institute, National Institutes of Health, Bethesda, MD 20892, USA; E-Mails: johnsonc@scripps.edu (C.H.J.); mannask@mail.nih.gov (S.K.M.); krauszk@intra.nci.nih.gov (K.W.K.); jessica.bonzo@lifetech.com (J.A.B.); 2 Developmental Therapeutics Program, SAIC-Frederick, Frederick National Laboratory for Cancer Research, Frederick, MD 21702, USA; E-Mail: divelbissr@mail.nih.gov; 3 Developmental Therapeutics Program, Division of Cancer Treatment and Diagnosis, National Cancer Institute-Frederick, Frederick, MD 21702, USA; E-Mail: hollingm@mail.nih.gov

**Keywords:** metabolomics, biomarker, MCF-7, breast cancer, xenograft

## Abstract

Global metabolomics analysis has the potential to uncover novel metabolic pathways that are differentially regulated during carcinogenesis, aiding in biomarker discovery for early diagnosis and remission monitoring. Metabolomics studies with human samples can be problematic due to high inter-individual variation; however xenografts of human cancers in mice offer a well-controlled model system. Urine was collected from a xenograft mouse model of MCF-7 breast cancer and analyzed by mass spectrometry-based metabolomics to identify metabolites associated with cancer progression. Over 10 weeks, 24 h urine was collected weekly from control mice, mice dosed with estradiol cypionate (1 mg/mL), mice inoculated with MCF-7 cells (1 × 10^7^) and estradiol cypionate (1 mg/mL), and mice dosed with MCF-7 cells (1 × 10^7^) only (n = 10/group). Mice that received both estradiol cypionate and MCF-7 cells developed tumors from four weeks after inoculation. Five urinary metabolites were identified that were associated with breast cancer; enterolactone glucuronide, coumaric acid sulfate, capric acid glucuronide, an unknown metabolite, and a novel mammalian metabolite, “taurosebacic acid”. These metabolites revealed a correlation between tumor growth, fatty acid synthesis, and potential anti-proliferative effects of gut microbiota-metabolized food derivatives. These biomarkers may be of value for early diagnosis of cancer, monitoring of cancer therapeutics, and may also lead to future mechanistic studies.

## 1. Introduction

Breast cancer is the most common cancer to affect women worldwide. In 2013, there will be 40,030 estimated deaths and 234,580 estimated new cases reported [1]. Early detection can dramatically improve the survival of the patient [2]. Current screening methods include mammography, magnetic resonance imaging, and clinical breast examination, but these methods all have limitations; the patient can still have a poor prognosis and not all breast cancer can be identified. Therefore, novel methods for detecting breast cancer at its earliest presymptomatic stages are needed. Screening for urinary biomarkers of breast cancer, discovered and validated by mass spectrometry (MS)-based metabolomics, could provide a rapid non-invasive method to detect presymptomatic breast cancer. A metabolic signature for breast cancer composed of one or more validated metabolites could indicate whether further screening is required and/or whether to initiate treatment regimens, thus, increasing survival rates and treatment success. In addition, tracking the reappearance of the biomarkers post-therapy could monitor the effectiveness of a treatment.

MS-based metabolomics is the ideal platform for biomarker discovery and the establishment of metabolic phenotypes. The majority of breast cancer studies carried out using metabolomics have been on tissue samples. The results of these studies showed perturbed choline metabolism, an association between increased levels of phospholipids, and poorer survival rates [3]. A limited number of metabolomic studies that have analyzed urine from breast cancer patients have shown metabolites related to oxidative damage, energy metabolism, amino acids, and the tricarboxylic acid cycle [4,5]. However, studies using human patients can be problematic due to inter-individual variation that results from environmental factors such as drugs, stress, diet, lifestyle, and geographical location. Genetics is also a major factor, and variation in microbiota adds another level of complexity when analyzing the urinary metabolome [6]. Xenograft mouse models can provide an ideal system to carry out human breast cancer metabolomic studies as this variation can be significantly reduced. Mice have distinct genetic backgrounds, and co-housing xenograft with control mice can reduce microbiota variation. Another advantage to using the xenograft mouse model is that the mice develop tumors at a similar pace, and therefore the metabolic response to tumor development can be monitored over time in a large sample size. In comparison, transgenic mice can be challenging as not all the mice carrying the transgene develop tumors, and those that do have a highly variable latency period. Therefore, the xenograft model is ideal for identifying biomarkers of human breast cancer.

Previously, metabolomic studies have been carried out in human xenograft mouse models to identify urinary biomarkers of cancer using glioblastoma multiforme cells [7], gastric adenocarcinoma cells [8], and lung adenocarcinoma cells [9]. These studies were primarily undertaken using nuclear magnetic resonance spectroscopy. Here, MS-based metabolomics was used to analyze urine collected from the MCF-7 human xenograft tumor model. The MCF-7 cell line used in this study is non-metastatic and estrogen receptor (ER) positive. Mice transplanted with this cell line and estradiol, developed large tumors within 10 weeks. The MCF-7 xenograft model has been used extensively to study breast cancer, in particular for testing potential therapeutics [10,11,12]. However, this is the first metabolomic study to use a xenograft mouse model of this cell line.

## 2. Results and Discussion

### 2.1. Animal Health

**Figure 1:**
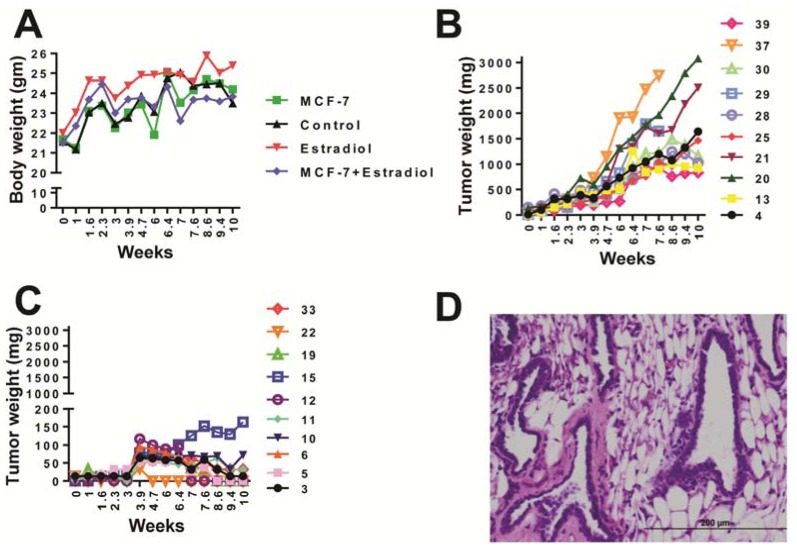
(**A**) Body weight of mice according to treatment group. One-way ANOVA between all four groups gave significant difference between estradiol *vs.* MCF-7 groups, and estradiol *vs.* MCF-7 plus estradiol group *p* = 0.0127, (**B**) Tumor weight for mice inoculated with estradiol and MCF-7 cells (mouse identification number on right axis), (**C**) Tumor weight for mice inoculated with MCF-7 cells only, (**D**) H&E staining of tumor taken from mouse number 21.

Over the course of 74 days, the body weights of mice in the study were recorded. Mice that received estradiol injections only had significantly higher body weights than mice receiving MCF-7 cell inoculations or MCF-7 inoculations plus estradiol injections (*p* = 0.0127) (Figure 1A). However, there was no difference between the control mice and mice that received MCF-7 cells and those receiving MCF-7 cells plus estradiol. Tumor formation was seen in all mice dosed with MCF-7 plus estradiol and the final tumor sizes ranged from 831 to 3,080 mg (Figure 1B). The average tumor size was 1700 ± 240 mg. Mice that received MCF-7 cells and no estradiol exhibited a small lump at the site of injection that mostly decreased over time, however, two mice had 72 mg and 163 mg tumors at 10 weeks (Figure 1C). H&E staining of a tumor from mouse number 21 (received MCF-7 cells and estradiol) revealed irregular duct shape with epithelial cell invasion (Figure 1D). One mouse from the estradiol only group was euthanized at 7.5 weeks due to estrogen toxicosis, and two mice from the MCF-7 cells plus estradiol group were euthanized at 8.5 weeks due to the occurrence of superficial tumor necrosis.

### 2.2. UPLC-ESI-QTOFMS-Based Metabolomics Analysis

**Figure 2:**
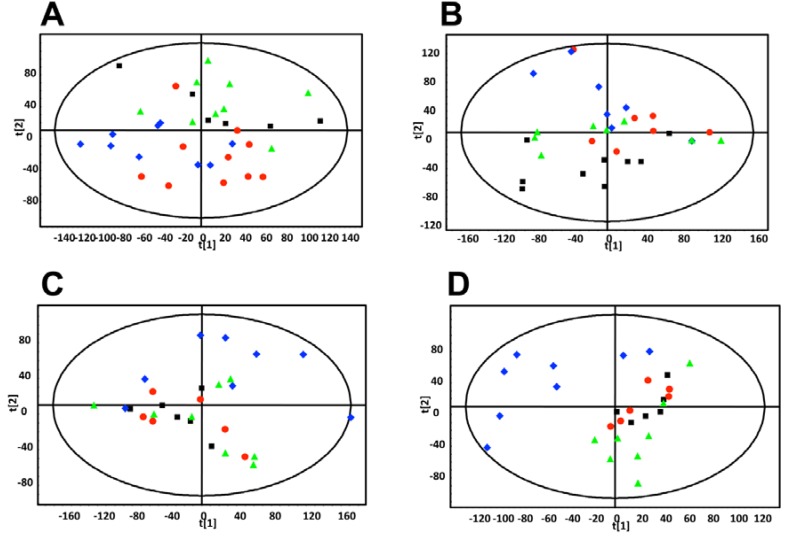
PCA scores plot of mouse urine samples analyzed by UPLC-ESI-QTOFMS ESI- (**A**) 1.5 weeks after dosing. (**B**) 7.5 weeks after dosing, and (**C**) 9.5 weeks after dosing.■ control, 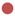 estradiol only, 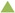 MCF-7 cells only, 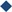 estradiol plus MCF-7 cells. These plots show increased intra-group clustering and inter-group separation at later time points. (**D**) PLS-DA scores plot of mouse urine samples analyzed by UPLC-ESI-QTOFMS ESI- 9.5 weeks after dosing.

**Figure 3:**
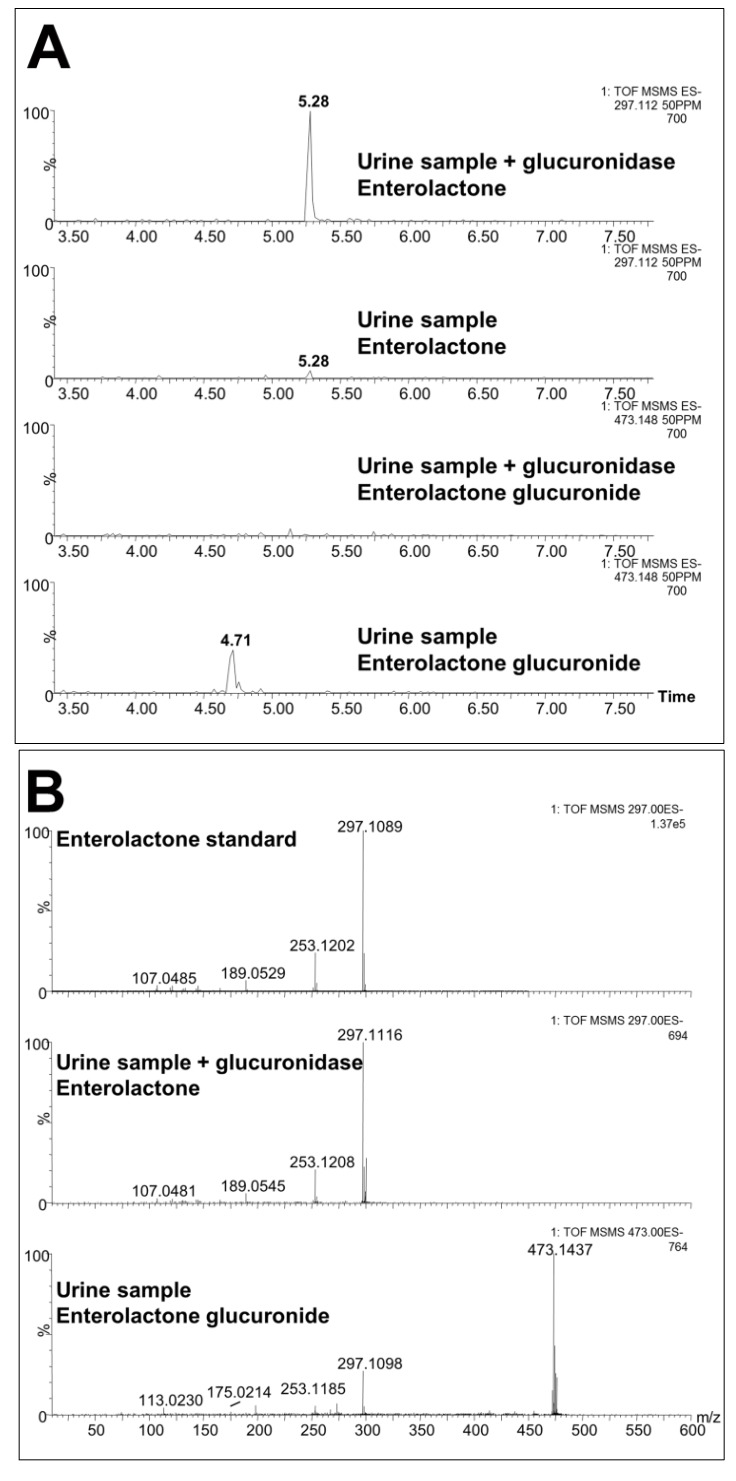
(**A**) Extracted mass chromatogram of enterolactone in urine treated with glucuronidase (upper chromatograph), enterolactone in urine before treatment with glucuronidase (upper middle chromatograph), enterolactone glucuronide in urine after treatment with glucuronidase (lower middle chromatograph), and enterolactone glucuronide in urine before treatment with glucuronidase (lower chromatograph). It can be seen how glucuronidase treatment deconjugates enterolactone glucuronide (peak at 4.71 min) to enterolactone (peak at 5.28 min). (**B**) Extracted tandem mass spectra of enterolactone standard 297.1 [M-H]^−^ (upper spectrum), enterolactone in urine treated with glucuronidase (middle spectrum) and enterolactone glucuronide in urine before treatment with glucuronidase (lower spectrum).

**Figure 4:**
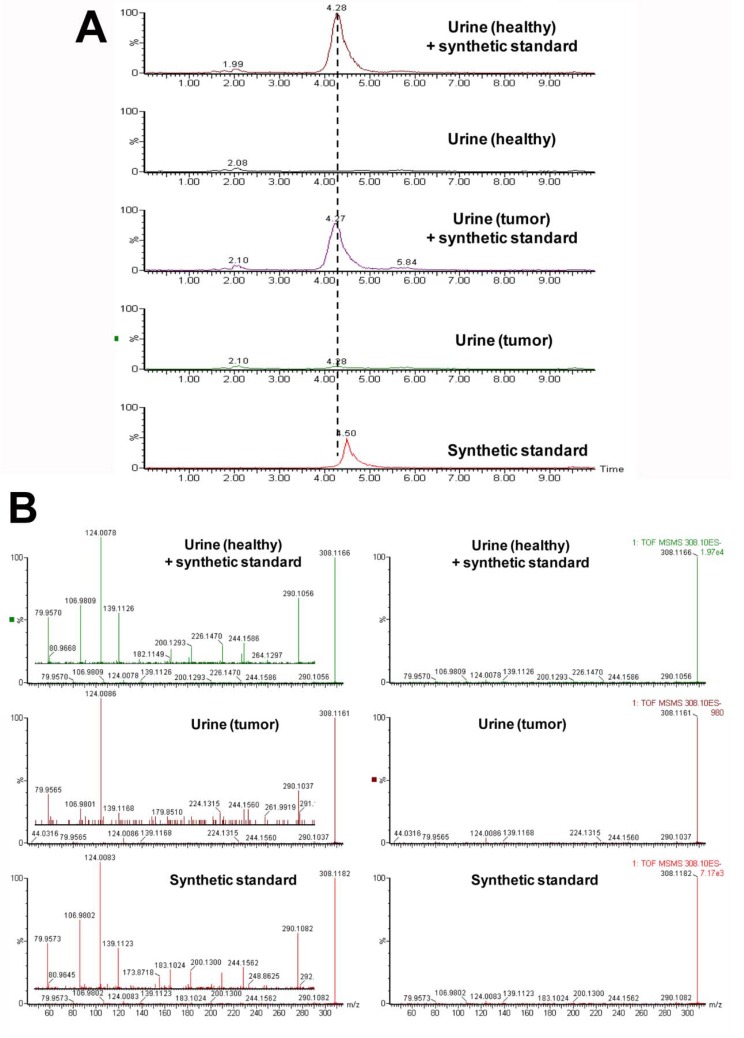
(**A**) Extracted mass chromatogram of the novel metabolite taurosebacic acid in urine 308.1167 [M-H]^−^. Retention time (RT) comparison and effect of spiking. (**B**) Tandem mass spectrum of taurosebacic acid in urine (healthy) spiked with synthetic standard, urine (tumor) and the synthetic standard alone.

**Figure 5:**
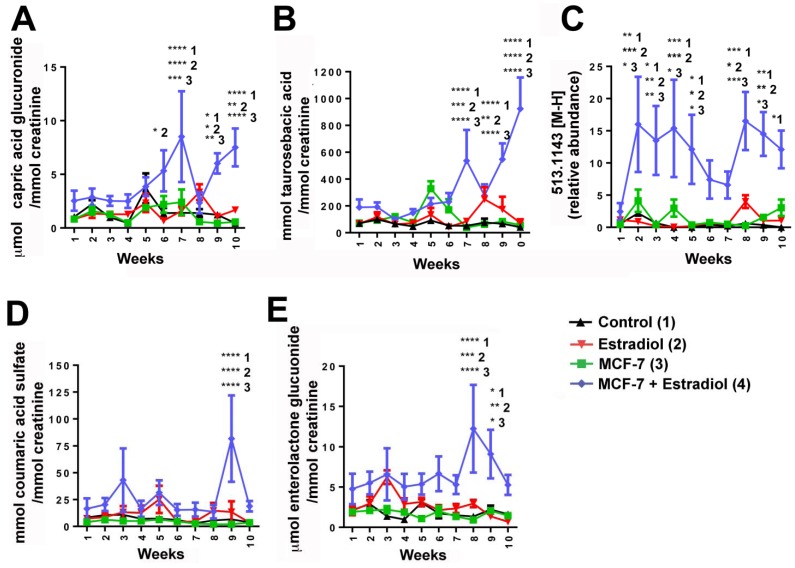
Mean concentrations of metabolites identified in mouse urine in each of the four mouse models: control (**1**), estradiol (**2**), MCF-7 (**3**) and MCF-7 + estradiol (**4**). (**A**) Capric acid glucuronide, (**B**) Taurosebacic acid, (**C**) Unknown metabolite 513.1443 [M-H]^−^, (**D**) Coumaric acid sulfate, and (**E**) Enterolactone glucuronide. Errors are SEM. Significance as determined by two-way ANOVA with Bonferroni correction, with comparisons of control (**1**), estradiol (**2**), and MCF-7 (**3**) made to MCF-7 + estradiol (**4**) treated mice annotated. Two-tailed *p*-value: ****p < 0.0001, ***p < 0.001, ** *p* < 0.01, * *p* < 0.05. Concentrations were calculated for (**A**), (**B**), (**D**) and (**E**) using standard calibration curves. Relative abundance was calculated for (**C**).

Weekly 24 h urine samples were collected from mice that were individually housed in metabolic cages. The samples were analyzed by ultraperformance liquid chromatography-electrospray ionization-quadrupole time-of-flight mass spectrometry (UPLC-ESI-QTOFMS) in electrospray ionization negative mode (ESI-). After pre-processing, and normalization to an internal standard, 3,207 features were recovered and the data were subjected to multivariate data analysis (MDA). Unsupervised PCA models were made comparing the four groups of mice at each time-point. Scores plots of urine samples collected from mice 1.5, 7.5, and 9.5 weeks post-inoculation revealed clustering of samples into their dosing groups at 1.5 weeks, but also some overlap between the groups (Figure 2A–C). At 7.5 weeks, the control and MCF-7 plus estradiol groups became more distinct. At 9.5 weeks, urine samples collected from mice inoculated with MCF-7 cells plus estradiol clustered together, and urine collected from the other mice clustered together as one group. Supervised PLS-DA analysis of this time-point further enhanced these groupings. Subsequent models were constructed comparing the four individual groups across the 10 week time-period. A number of OPLS-DA models were also constructed comparing combinations of groups; for example MCF-7 plus estradiol compared to all other groups, MCF-7 plus estradiol compared to estradiol only, control compared to MCF-7 only. Analysis of trends across the time-periods and in all samples revealed metabolites that were associated with tumor development; five metabolites were significantly correlated to tumor growth at various time points (Table 1). These metabolites were initially subjected to tandem MS for identification, with three of the metabolites identified as sulfate and glucuronide conjugates. This was confirmed by deconjugation with sulfatase and glucuronidase, and analysis by tandem MS with comparison against authentic standards; an example can be seen on Figure 3 for enterolactone glucuronide. Thus, of the five metabolites correlated to tumorigenesis, two were positively identified as enterolactone glucuronide and coumaric acid sulfate (Table 1). Another was tentatively identified as capric acid glucuronide after deconjugation with glucuronidase and comparison to an authentic standard. A novel metabolite was identified as mono-taurosebacic acid after comparison to a synthesized standard (Figure 4A,B). The remaining metabolite could not be positively identified but had fragments matching estradiol and estradiol sulfate, as revealed using the METLIN database [13]. All five metabolites were quantitated for validation and to obtain their relative concentrations (Figure 5A–E). Conjugated standards were not commercially available therefore enterolactone, capric acid, and coumaric acid were used to make standard curves for relative quantitative measurements. All concentrations were normalized to creatinine to account for differences in glomerular filtration rate and urine dilution.

Enterolactone glucuronide was increased in the mice bearing MCF-7 tumors, 9.1-fold (eight weeks) and 4.1-fold (nine weeks) compared to control, 4.1-fold (eight weeks) and 6.7-fold (nine weeks) compared to estradiol-supplemented mice only, and 13.0-fold (eight weeks) and 4.5-fold (nine weeks) compared to MCF-7 only groups respectively. Coumaric acid sulfate was also significantly increased 12.0-, 6.0-, and 40-fold at nine weeks (with same comparison respectively). Capric acid glucuronide and mono-taurosebacic acid were significantly upregulated in the urine of mice bearing tumors at six weeks, (capric acid glucuronide only), seven weeks, nine weeks, and 10 weeks. The largest fold increases were at seven weeks for capric acid 6.0, 5.3, and 3.5-fold, and at 10 weeks for taurosebacic acid at 22.1, 12.6, and 16.0-fold, in mice bearing tumors, compared to control, estradiol only and MCF-7 only mouse groups respectively. The unknown metabolite was increased significantly at 2–5 weeks and 8–10 weeks, with fold increases ranging from ~5.0-400.0-fold, however this metabolite decreased in significance at 10 weeks.

**Table 1:** Metabolites associated with tumor development observed in ESI-.

Rank	Metabolite	Retention Time (min)	Identification	Confirmed / putative	Mass error (ppm)	Fragments
1	347.1709	5.71	Glucuronidated metabolite (potentially capric acid glucuronide)	Putative	0.8	175.0243, 171.1389, 113.0237
2	308.1167	3.52	Taurosebacic acid	N/A	N/A	124.0075, 106.9808, 79.9569
3	473.1450	4.73	Enterolactone glucuronide	Confirmed against standard and by deconjugation	0.4	297.1118, 253.1202, 189.0542, 175.0235, 165.0532, 113.0241
4	513.1443	4.71	Unknown	N/A	N/A	351.0900, 271.1331, 237.1263
5	242.9967	2.18	Coumaric acid sulfate	Confirmed against standard and by deconjugation	1.6	163.0385, 119.0496, 79.9582

## 3. Experimental Section

### 3.1. Compounds

Creatinine, coumaric acid, capric acid, and enterolactone, were all obtained from Sigma Aldrich (St. Louis, MO, USA). Estradiol Cypionate Injection, USP was purchased from Henry Schein (Melville, NY, USA). All other chemicals were of the highest purity grade.

### 3.2. Synthesis of Mono-Taurosebacic Acid

In brief, 200 mg sebacic acid, 150 mg *N*-hydroxybenzotriazole, 500 mg (2-(6-Chloro-1H-benzotriazole-1-yl)-1,1,3,3-tetramethylaminium hexafluorophosphate), and 120 mg taurine were added to an oven-dried 100 mL round-bottom flask. Dry acetonitrile (5 mL) was added and stirred for 15 min. *N*, *N*-diisopropylethylamine (300 μL) was added drop-wise into the slurry. The reaction mixture was stirred overnight at room temperature, acidified, and extracted with ethylacetate. The aqueous layer containing the product was evaporated to dryness.

### 3.3. MCF-7 Xenograft

MCF-7 cells, obtained from the DCTD Repository (Frederick, MD, USA), were cultured in RPMI Medium 1640 (Life Technologies, Carlsbad, CA, USA) supplemented with 10% fetal bovine serum and 2 mM L-glutamine. The cells were maintained in a humidified incubator at 37 °C in the presence of 5% CO_2_. For implantation, the cells were harvested by trypsinization, washed with saline, assessed for viable cell count by trypan blue dye exclusion, and diluted to the final working concentration. Athymic *nu*/*nu*/NCr (n = 40) were obtained from the National Cancer Institute Animal Production Program (Frederick, MD, USA). At six weeks of age, they were randomized into four groups of 10. Group one was a control group, group two received estradiol cypionate, group three received estradiol cypionate and MCF-7 cells, and group four received MCF-7 cells without estradiol cypionate supplementation. Estradiol cypionate (1 mg/mL) was administered as a subcutaneous injection between the shoulder blades once every seven days for the duration of the study. MCF-7 cells (1 × 10^7^) were surgically implanted into the 4th mammary fat pad on day one using standard procedures with the mice anesthetized using isoflurane by inhalation.

Tumor size was monitored using digital calipers (length and width) and body weight was recorded weekly after removal from metabolic cages. Tumor weight was calculated using the formula for a prolate ellipsoid and assuming a density of 1.0 g/cm^3^ [14]. The mice were acclimated to and maintained on an AIN-93M diet (Dyets, Inc., Bethlehem, PA, USA) throughout the study. Autoclaved tap water was given *ad libitum*. At 74 days post-tumor inoculation, the mice were euthanized and the tumors collected. Any mice with evidence of estradiol related toxicity or tumor abnormalities were euthanized at the time of discovery. Tumors were fixed in 10% neutral-buffered formalin, processed through paraffin, sectioned, and stained with hematoxylin and eosin (H&E). All studies were conducted in an Association for Assessment and Accreditation of Laboratory Animal Care International (AAALACi)-accredited facility in compliance with the Public Health Service Guidelines for the Care and Use of Animals in Research.

### 3.4. Urine Collection and Preparation for UPLC-ESI-QTOFMS

Mice were acclimated to metabolic cages using three cycles of 24 h on and 48 h off prior to the start of sample collections. Starting three days after MCF-7 injection, the mice were regularly removed from group housing and placed into individual metabolic cages. After 24 h the mice were returned to their group housing and urine collected, the volume of urine was recorded and samples were stored at −80 °C. The urine samples were thawed, 50 μL was added to microcentrifuge tubes containing 50 μL ice cold acetonitrile: water (50:50 *v*/*v*) and 5 μM chlorpropamide at 4 °C. The samples were vortexed for 1 min and centrifuged at 14,000 × g for 20 min at 4 °C to remove proteins and particulates. The supernatants were transferred to UPLC vials. A pooled sample was also made for quality control containing 5 μL of each sample.

### 3.5. UPLC-ESI-QTOFMS Analysis

The samples were randomized and analyzed by UPLC-ESI-QTOFMS as described previously [15]. In brief, the following mobile phase linear gradient consisting of 0.1% formic acid (A) and acetonitrile containing 0.1% formic acid (B) was used with a flow rate of 0.5 mL/min; 98% A for 0.5 min to 80% A at 4.0 min to 95% B at 8 min. The column was washed with 100% B for 1 min then equilibrated with 98% A for 1 min before subsequent injections. Samples were injected onto a reverse-phase 50 × 2.1 mm ACQUITY^®^ 1.7 μL C18 column (Waters Corp, Milford, MA, USA) using an ACQUITY^®^ UPLC system (Waters Corp, Milford, MA, USA). A water blank and pooled sample was injected after every five samples consecutively. Mass spectrometry was performed on a Waters^®^ QTof-Premier^TM^-MS operating in negative ESI mode.

### 3.6. Multivariate Data Analysis and Biomarker Identification

The mass spectral data were centroided, integrated, and deconvoluted to generate a multivariate data matrix using MarkerLynx^®^ (Waters Corp, Milford, MA, USA). Peak picking, alignment, deisotoping, and integration were performed automatically by the software with the following parameters: mass tolerance = 0.05 Da, peak width at 5% height = 1 s, peak-to-peak baseline noise 10, intensity threshold = 100 counts, mass window = 0.05 Da, retention time window = 0.20 min and noise elimination level = 10. The data were also normalized to the total ion current (TIC) chromatogram by the Markerlynx program. The raw data were then transformed into a multivariate matrix containing aligned peak areas with matched mass-to-charge ratios and retention times. The data were normalized to the peak area of the internal standard chlorpropamide (5 μM), which appeared at a retention time of 5.3 min, 275.024 [M-H]^−^ (deprotonated molecular ion) and exported into SIMCA-P+ software (Umetrics, Kinnelon, NJ, USA). The ESI- data were Pareto-scaled to increase the importance of low abundance ions without significant amplification of noise, and analyzed by principal components analysis (PCA), partial least squares discriminant analysis (PLS-DA), and orthogonal projection to latent structures discriminant analysis (OPLS-DA). For identification of biomarkers specific to breast cancer OPLS-DA models were constructed, ions with a correlation (pcorr) above 0.8, and peak area above 100, were subjected to tandem MS. Further confirmation of identity was then carried out by repeating the tandem MS fragmentation using authentic standards at 100 μM in water and in urine.

### 3.7. Deconjugation of Sulfated and Glucuronidated Metabolites

A number of conjugated metabolites were seen in the urine samples. For each deconjugation reaction, 40 U sulfatase (from *Helix Pomotia*, Sigma Aldrich (St. Louis, MO), USA) or β-D-glucuronidase were dissolved in 50 μL 0.2% (*w*/*v*) sodium chloride solution and added to a 1.5 mL microcentrifuge tube containing 50 μL urine and 400 μL pH 5.0 (sulfatase) or pH 3.8 sodium acetate buffer 200 mM. Controls were also made in tandem as above but without sulfatase. *P*-nitrocatechol sulfate was used as a positive control for the sulfatase deconjugation reaction and phenolphthalein β-D-glucuronide (PPTGlu) for glucuronidase. The mixtures were incubated overnight at 37 °C. Acetonitrile (500 μL) was added and the samples were vortexed and centrifuged for 15 min at 14,000 rpm and 4 °C. The supernatant was concentrated to a final volume of 250 μL for 3 h in a Savant Speedvac^®^ (Thermo Scientific, Waltham, MA, USA). Chlorpropamide (5 μM) was added as an internal standard and 5 μL of each solution was injected onto the UPLC-ESI-QTOFMS for analysis by tandem MS fragmentation and comparison to standards where available.

### 3.8. Quantitation

Biomarkers were quantitated using an Acquity^®^ UPLC H-class coupled to a XEVO^®^G2 QTOFMS with Quantof™ technology (Waters Corp, Milford, MA, USA) as described previously [16]. Standard calibration curves of 0 to 35 μM were made for creatinine, enterolactone glucuronide, coumaric acid sulfate, and capric acid sulfate using the following commercially available authentic standards; creatinine, enterolactone, coumaric acid, and capric acid. A relative abundance was calculated for the unknown metabolite. Urines were diluted 1:2 in water; an internal standard of 5 μM chlorpropamide was added to each sample, with a final concentration of 5 μM. The chromatographic conditions used were as listed above except that an additional 2 min equilibration step was needed with the H-class system. The XEVO^®^G2 was operated in ESI- with a capillary voltage of 3,000 V and a sampling cone voltage of 30 V. The desolvation and cone gas flow were set to 850 and 50 L/H, respectively. The desolvation temperature was set to 450 °C and the source temperature was 150 °C. Data were acquired in centroid mode from 50 to 850 *m/z.* The samples were quantitated using TargetLynx^®^ (Waters Corp, Milford, MA, USA) software by integrating peak areas of extracted ion chromatograms.

### 3.9. Statistics

All concentrations were expressed as mean ± standard error of the mean (SEM) 2-way ANOVA with a Bonferroni multiple comparisons test using GraphPad Prism 4 software (GraphPad Software, Inc., La Jolla, CA, USA). A *p*-value < 0.05 was considered statistically significant.

## 4. Conclusions

Global metabolomics analysis of MCF-7 xenograft mice revealed five urinary metabolites highly correlated to tumor growth. From weeks six and seven of tumor development, two metabolites increased in concentration to similar significance and fold change (when compared to control groups) for the rest of the time-course; capric acid glucuronide (decanoic acid glucuronide) and a novel mammalian metabolite; mono-taurosebacic acid (taurine-conjugated decanedioic acid). At week eight the concentration of both metabolites fell to basal levels, the reason for this is undetermined. Unconjugated sebacic acid is a normal mammalian urinary metabolite, but increases have been used to diagnose patients with multiple acyl-CoA-dehydrogenase deficiency (MADD) or glutaric aciduria type II (GAII) [17,18]. These diseases occur due to deficiencies in electron transfer flavoprotein or electron transfer flavoprotein ubiquinone oxidoreductase. Taurine conjugation of sebacic acid in these tumor-bearing mice indicated an increased need for sebacic acid excretion. Excess sebacic acid could also potentially arise from increased fatty acid synthesis, a known cancer mechanism. It is possible that sebacic acid could be produced from ω-oxidation of capric acid with a concomitant production of NADH, serving as an alternative energy source. This indicates aberrant energy metabolism in these fat-rich tissues. Thus glucuronidation of capric acid and taurine-conjugation of sebacic acid reveal an increased need for excess capric acid excretion.

At week eight of the study, enterolactone glucuronide was significantly upregulated in tumor-bearing mice. Enterolactone is a phytoestrogen and biologically active enterolignan formed from matairesinol by the gut microbiota. It is found at high concentration in seeds, cereals, and grains. Enterolactone can be reabsorbed in the intestine to undergo enterohepatic circulation; the gut microbiota are thus a major determinant of enterolactone absorption [19]. Many breast cancer studies have focused on enterolactone as a potential anti-proliferative agent. Its structure is similar to that of 17β-estradiol, and has binding affinity to the ERa. It also mediates ER transcription [20]. Increased urinary enterolactone glucuronide in the tumor-bearing mice could be due to two responses. Firstly 17β-estradiol may have a higher affinity than enterolactone for the ER, thus, enterolactone cannot bind, and secondly gut microbiota-tumor feedback may prevent re-entry of enterolactone glucuronide into the enterohepatic circulation. The preference of estradiol over enterolactone for the ER could ensure that enterolactone will not perform its anti-proliferative and apoptotic effects, thus tumor growth is increased in these mice. *In vitro* studies have shown that enterolactone can inhibit tumor growth in MCF-7 cell lines, with high concentrations of enterolactone inhibiting cellular proliferation by 97% compared to 60% by matairesinol; however at low concentrations (1 × 10^−5^ to 1 × 10^−7^ mol/L) a stimulatory effect was seen [21]. *In vivo* studies with MCF-7 xenograft models have shown that mice fed a soy protein diet had increased tumor growth. When these mice were also supplemented with flax seed, tumor growth was significantly attenuated with increased apoptosis, and inhibition of cellular proliferation [22]. Other MCF-7 xenograft studies have shown decreased tumor sizes in mice fed a diet supplemented with sesamin (a plant phytoestrogen that is also metabolized to enterolactone) [23], in mice with high estradiol levels that were supplemented with flax seed oil [24], and in mice fed a flax seed diet or given enterolactone injections [25].

As mentioned above, there may be some interplay between the gut microbiota and tumors, and there could be a role for certain gut microbiota species in tumor development in relation to enterolactone formation. Gnotobiotic rats were colonized with four strains of lignin-converting bacteria, fed a lignin-containing diet, and induced with breast cancer. Compared to germ-free controls, decreased tumor size, proliferation, and increased apoptosis were observed [26].

The presence of enterolactone glucuronide and its possible competition with estradiol for the ER may also explain the presence of another metabolite that has an *m/z* of 513.1443 [M-H]^−^. While it was not possible to confirm the identity of this metabolite, MS fragmentation revealed that it may be a metabolite of estradiol sulfate, as a result of dosing the mice with estradiol cypionate. Mice that received estradiol cypionate only did not excrete this metabolite at these levels indicating both estradiol cypionate and tumorigenesis are required for increased production of this metabolite. However, 513.1443 *m/z* ion was not confirmed as estradiol sulfate and it is possible that it is a different metabolite structurally similar to estradiol sulfate.

Seven weeks into the study, another metabolite was significantly correlated with tumor development; coumaric acid sulfate. Coumaric acid is a hydroxycinnamic acid found in edible plants, wine and vinegar. Hydroxycinnamic acids have been postulated to act as antioxidants, inhibiting oxidative stress related to cancer development [27,28]. They have also been shown to have biological activity against tumor cells, modulating apoptosis and cell-cycle regulation. Many studies have been carried out using derivatives of hydroxycinnamic acid such as ferulic and caffeic acids and shown them to be anti-proliferative and cytotoxic when dosed to ER positive and negative breast cancer cell lines [28,29]. It has also been shown that cinnamic acids can inhibit 17β-hydroxysteroid dehydrogenase (17β-HSD) type 1 [30]. This enzyme converts estrone to estradiol, and thus can have an effect on ER positive breast cancer. Therefore, both coumaric acid and enterolactone, essentially food derived compounds, can potentially have anti-proliferative effects on ER positive breast cancer.

Global metabolomics analysis of urine collected from a xenograft mouse model of MCF-7 breast cancer has enabled the identification of five urinary metabolites, including a novel mammalian metabolite mono-taurosebacic acid. All five metabolites were highly correlated to tumorigenesis. Data from this study also suggested that gut microbiota play a large role in metabolism of these metabolites when cancer cells are present, enhancing the production of anti-proliferative metabolites through a gut microbiota-tumor feedback. Increases in C-10 fatty acids were also seen indicating an aberration to fatty acid synthesis. This study shows the value of carrying out untargeted metabolomics with xenograft mouse models of cancer to find novel downstream metabolites of tumorigenesis.
